# Three new species of *Begonia* endemic to the Puerto Princesa Subterranean River National Park, Palawan

**DOI:** 10.1186/s40529-015-0099-1

**Published:** 2015-07-24

**Authors:** Rosario Rivera Rubite, Mark Hughes, Patrick Blanc, Kuo-Fang Chung, Hsun-An Yang, Yoshiko Kono, Grecebio J D Alejandro, Llogene B De Layola, Arthur Gregory N Virata, Ching-I Peng

**Affiliations:** 1 Department of Biology, College of Arts and Sciences, University of the Philippines Manila, Padre Faura, Manila, 1000 Philippines; 2 Royal Botanic Garden Edinburgh, 20a Inverleith Row, Edinburgh, EH3 5LR UK; 3 CNRS, 3 rue Michel-Ange, 75794 Paris, France; 4 School of Forestry and Resource Conservation, National Taiwan University, Taipei, 106 Taiwan; 5 Biodiversity Research Center, Academia Sinica, Taipei, 115 Taiwan; 6 College of Science and Research Centre for the Natural and Applied Sciences, University of Santo Tomas, España, Manila, 1015 Philippines

**Keywords:** Limestone, Endemic, New species, Conservation

## Abstract

**Background:**

*Begonia* is a mega-diverse genus of flowering plants prone to generating micro-endemic species, especially on limestone habitats. During fieldwork in the Puerto Princesa Subterranean River National Park, Palawan (Philippines), three species were encountered which did not match any previously described from the region.

**Results:**

Following morphological, anatomical, molecular phylogenetic and cytological investigation a hypothesis of three new species is supported. The three new species belong to a clade endemic to Palawan and Borneo.

**Conclusions:**

The limestone habitats in the Puerto Princesa Subterranean River National Park environs support a unique flora. The description of three new species from a small area within the park demonstrates how much remains to be discovered there, and the importance of its continued protection.

**Electronic supplementary material:**

The online version of this article (doi:10.1186/s40529-015-0099-1) contains supplementary material, which is available to authorized users.

## Background

The Philippines is a biodiversity hotspot, with an exceptional concentration of endemic species undergoing exceptional loss of habitat (Myers et al. 2000). Some of the most extensive areas of intact forest in the country are found in Palawan, where there are reasons for optimism due to the implementation of conservation through devolved local government and civil society groups (Posa et al. 2008). Puerto Princesa Subterranean River National Park (PPSRNP) is managed by the City Government of Puerto Princesa in partnership with the Department of Environment and Natural Resources (DENR), and is the first national park in the Philippines to be managed at this level. It was designated a World Heritage Site in 1999 (World Heritage Commitee 2000), and voted as one of the *New 7 Wonders of Nature*, boosting tourism as much as 300% (Fitzgerald 2011, 2012). Palawan as a whole is designated by UNESCO as a Biosphere Reserve, and by Birdlife International as an Important Bird and Biodiversity Area (BirdLife International 2015).

The PPSRNP contains a spectacular karst limestone landscape and eight different forest formations; forest on ultramafic soil, forest on limestone soil, montane forest, freshwater swamp forest, lowland evergreen tropical rainforest, riverine forest, beach forest, and mangrove forest. This mix of forest types and substrates means the PPSRNP harbours a very rich flora, but much remains to be discovered. During recent fieldwork in the PPSRNP we found three new species of *Begonia* within a distance of only a few hundred meters, adding these distinct species to the 14 known *Begonia* species already recorded from Palawan (Hughes and Coyle 2009; Hughes et al. 2010, 2011). Based on morphology, chromosome cytology and molecular phylogenetic analysis we place the three new species in *Begonia* sect. *Baryandra*. Although the species occur very close to one another at altitudes of less than 50 m, they grow in mutually exclusive habitats. The first species *Begonia taraw* C.-I Peng, R. Rubite & M. Hughes is located around the mouth of the underground river, growing tenaciously on the vertical limestone cliffs. Nearby in karst forest growing in rock crevices is the second species *Begonia hughesii* R. Rubite and C.-I Peng. The third species, *Begonia tagbanua* M. Hughes, C.-I Peng & R. Rubite, grows on clay soil banks inside the forest.

## Methods

### Chromosome cytology

Somatic chromosomes of *B. taraw* (*Ching*-*I Peng 23463, 23464*), *B. hughesii* (*Ching*-*I Peng 23466, 23475*), *B. tagbanua* (*Ching*-*I Peng 23471*) were examined using root tips. The procedures of pre-treatment, fixation and staining for chromosome observations followed Peng et al. (2014). Voucher specimens are deposited in HAST.

### Cryo scanning electron microscopy

Fresh leaves of *B. taraw*, *B. hughesii* and *B. tagbanua* were dissected and attached to a stub. The samples were frozen with liquid nitrogen slush, then transferred to a sample preparation chamber at −160°C and etched for 15 min at −85°C. After etching, the temperature reached −130°C for sample fracturing and coating. After coating, the samples were transferred to the SEM chamber and observed at −190°C with a cryo scanning electron microscope (FEI Quanta 200 SEM/Quorum Cryo System PP2000TR FEI).

### Phylogenetic analyses

A total of 33 species from *Begonia* sect. *Baryandra* was sampled, including the 3 new species from Palawan (each represented by two individuals) and a further 5 from Palawan (*Begonia blancii*, *B. cleopatrae*, *Begonia suborbiculata*, *B. wadei*, *B. woodii*). In addition three species of *Begonia* sect. *Reichenheimia* were sampled, with two species of *Begonia* sect. *Coelocentrum* as an outgroup based on the tree topology in Thomas et al. (2012). Vouchers and genbank accession numbers are in Additional file 1: Table S1. *Begonia* sect. *Baryandra* has recently been recircumscribed and confirmed as monophyletic (Rubite et al. 2013). Total genomic DNA was extracted from young leaves and buds using DNeasy Plant Mini Kits (Qiagen, USA). Four chloroplast non-coding regions (*ndh*A intron, *ndh*F–*rpl*32 spacer, *rpl*32–*trn*L spacer, *trn*C–*trn*D spacer) were amplified as in Hughes et al. (2015) with the PCR primer sequences from Demesure et al. (1995) and Thomas et al. (2011). Forward and reverse reads for all regions were assembled and aligned using Geneious Pro (Biomatters, New Zealand), with the alignments being subsequently manually edited in BioEdit 7.1.3 (Hall 1999); inversions were offset and the final alignment was 6905 bases long, with 39 bases excluded due to alignment uncertainty. The optimal model of DNA sequence evolution was assessed with jModeltest 2.1.3 (Posada et al. 2012) using the Aikake Information Criterion (AIC) to estimate the model with the closest fit to the data. The GRT + G + I model was the most probable (AIC weight of 0.98). Bayesian phylogenetic analysis was carried out using MrBayes 3.2.1 (Ronquist et al. 2012), treating the dataset as a single partition. The analysis consisted of two runs with four chains each, run for 10,000,000 generations with a sample tree taken every 1,000. The effective sample size was >5,600 for all model parameters, and the average standard deviation of split frequencies was 0.0016. The proportion of successful state exchanges between adjacent chains ranged between 0.62 and 0.68 for both runs. Convergence between runs and attainment of stationarity was runs was further comfirmed by examining parameter distrubutions in Tracer v1.6 (Rambaut et al. 2014). The first 25% of sampled trees were discarded as burn-in, and the remainder summarised as a maximum clade credibility tree visualised using FigTree v.1.4.0 (Rambaut 2009).

## Results

### Chromosome cytology

**Figure 1:**
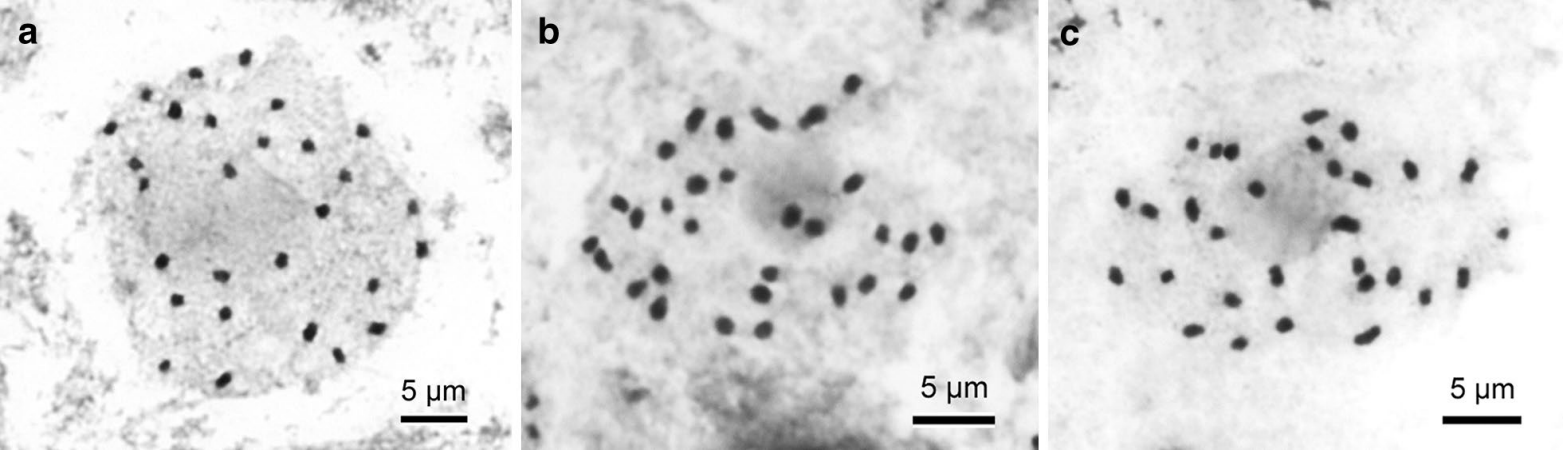
Somatic chromosomes at metaphase of *Begonia*. **a** *B. taraw* (2n = 28 [0.9–1.6 µm], Peng 23464); **b** *B.hughesii* (2n = 30 [1.0–2.1 µm], Peng 23466); **c** *B. tagbanua* (2n = 30 [0.9–2.0 µm], Peng 23471).

Recently, Philippine *Begonia* species belonging to section *Diploclinium* were transferred to section *Baryandra*, based on molecular analysis (Rubite et al. 2013). Among 47 species in sect. *Baryandra*, chromosome numbers were previously reported for five species, namely *B. blancii* (2*n* = 30, Hughes et al. 2011); *B. fenicis* (2*n* = 26, Oginuma and Peng 2002; 2*n* = 56, Kokubugata and Madulid 2000); *B. parva* (2*n* = 36 + 2f, Legro and Doorenbos 1969); *B. nigritarum* (2*n* = 44, Doorenbos et al. 1998); and *B. suborbiculata* (2*n* = 30, Hughes et al. 2011). Somatic chromosomes at metaphase of the three new species are reported here for the first time: 2*n* = 28 for *Begonia taraw* (Figure 1a); 2*n* = 30 for *B. hughesii* (Figure 1b) and *B*. *tagbanua* (Figure 1c).

### Leaf anatomy and vestiture

#### *Begonia taraw*

**Figure 2:**
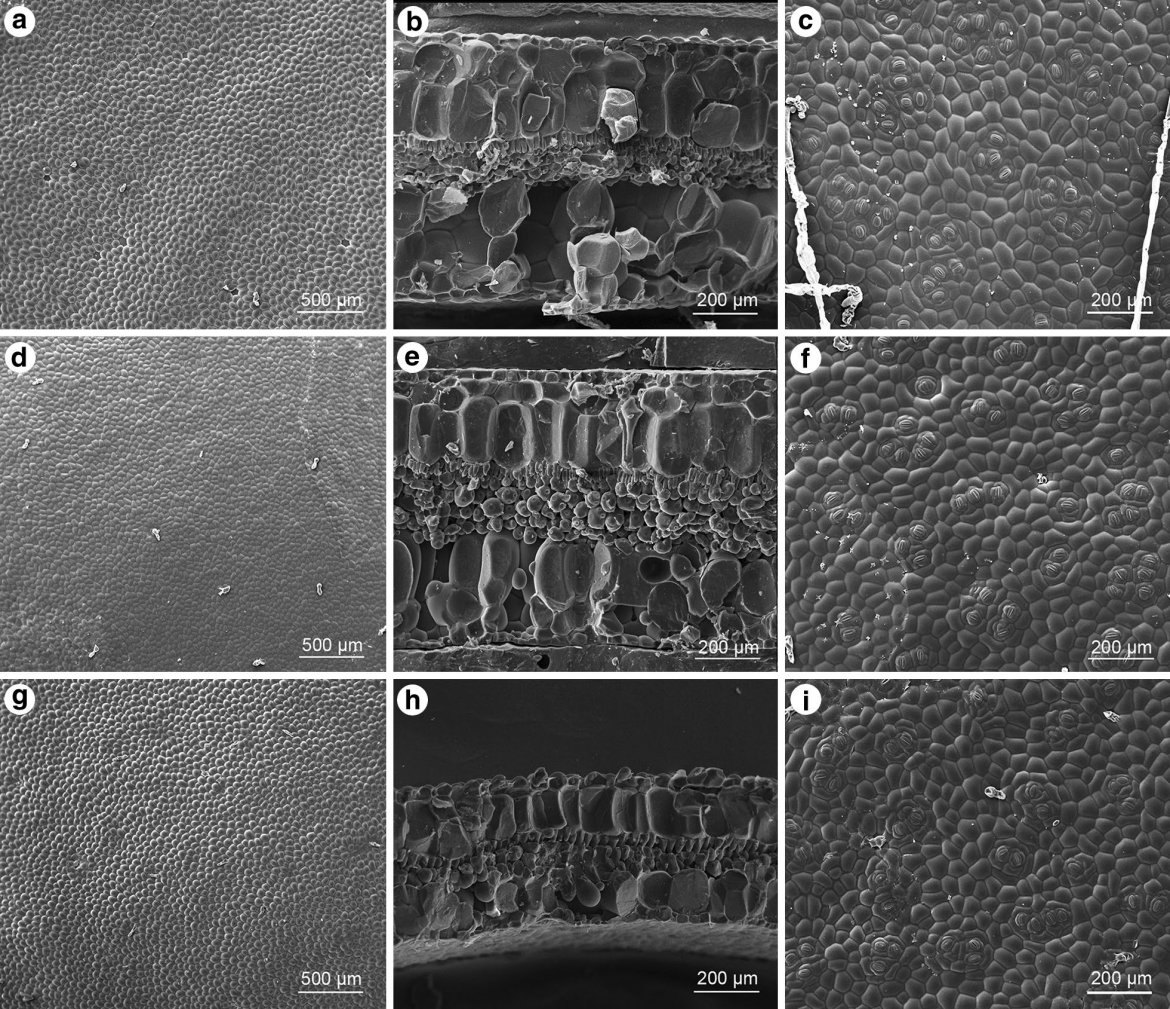
Leaf SEM microphotographs of *Begonia*. **a**–**c** *Begonia taraw*; **d**–**f** *B. hughesii*; **g**–**i** *B. tagbanua*; **a**, **d**, **g**, adaxial surface; **b**, **e**, **h**, cross section; **c**, **f**, **i**, abaxial surface.

Adaxial surface nearly glabrous, epidermis cells slightly bullate (Figure 2a, b); cross section ca. 820 μm thick; epidermis single-layered on both surfaces, hypodermis 2-layered; palisade parenchyma cells 1-layered; spongy parenchyma cells ca. 3-layered (Figure 2b); abaxial surface with glandular hairs and multiseriate trichomes, stomata in clusters of 3–7, helicocytic, flat, subsidiary cells 3 (Figure 2c).

#### *Begonia hughesii*

Adaxial surface with glandular trichomes (Figure 2d); cross section ca. 870 μm thick; epidermis single-layered on both surfaces, hypodermis 2-layered; palisade parenchyma cells 1-layered; spongy parenchyma cells ca. 4-layered (Figure 2e); abaxial surface with glandular trichomes, stomata single or 2–5 clustered, helicocytic, flat, subsidiary cells 3 (Figure 2f).

#### *Begonia tagbanua*

Adaxial surface with conoidal cells and glandular trichomes (Figure 2g, h); Cross section ca. 490 μm thick; epidermis single-layered on both surfaces, hypodermis 1-layered; palisade parenchyma cells 1-layered; spongy parenchyma cells ca. 2-layered (Figure 2h); abaxial surface with glandular trichomes, stomata 2–4 clustered, helicocytic, flat, subsidiary cells 3 (Figure 2i).

### Phylogenetic relationships

**Figure 3:**
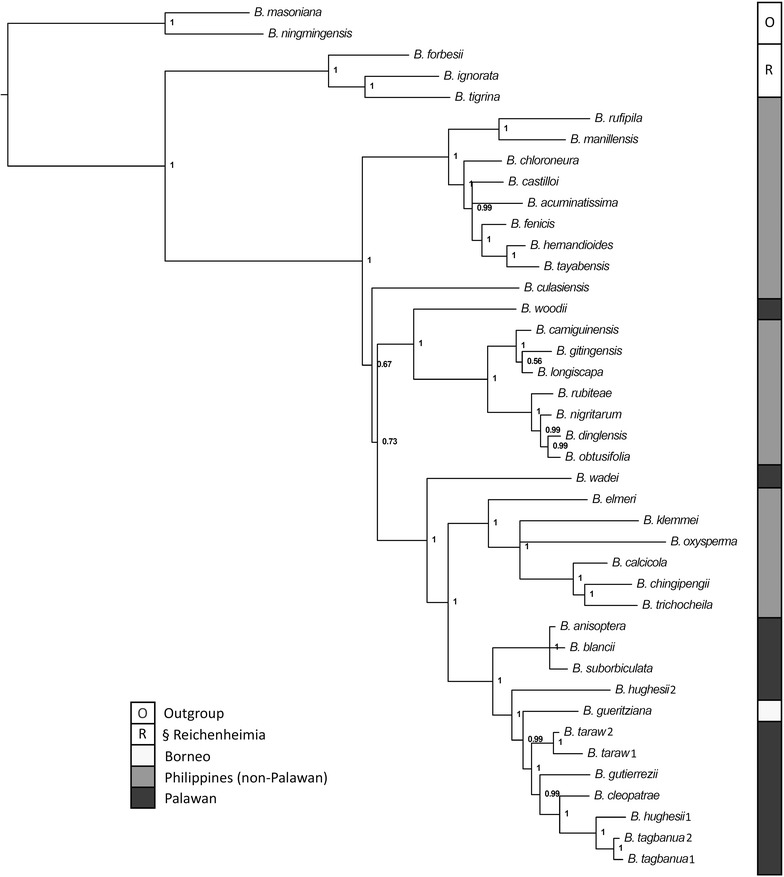
Phylogeny of *Begonia* sect. *Baryandra*. Generated from a Bayesian analysis of chloroplast DNA sequence data, the 50% majority-rule consensus tree is shown. The *figures* at the nodes are clade support values (posterior probabilities).

The three new species *B. hughesii*, *B. tagbanua* and *B. taraw* are strongly supported as being members of *Begonia* sect. *Baryandra*, and belong to a clade mostly endemic to Palawan (Figure 3). *Begonia hughesii* is not monophyletic according to its chloroplast genotype, whereas *B. tagbanua* and *B. taraw* are supported as monophyletic whilst having some within-species polymorphism as shown by the non-zero branch lengths. The two samples of *Begonia hughesii* were taken from different localities; sample 1 (*Peng 23466*) was collected in littoral forest near Sabang, and sample 2 (*Peng 23475*) was collected at Ugong Rock.

### Taxonomic treatment

**Figure 4:**
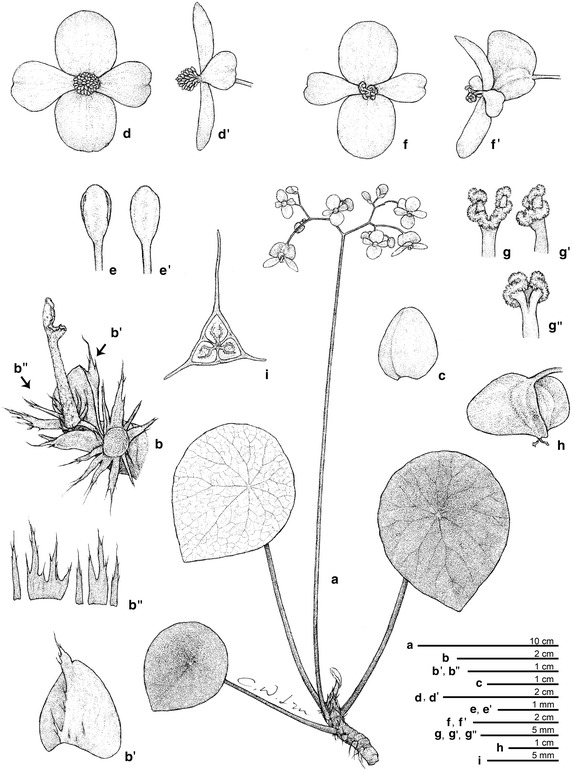
*Begonia taraw* C.-I Peng, R. Rubite, & M. Hughes. **a** Habit; **b** showing the base of petiole; **b’**, stipule; **b”**, fleshy hairs fused into a ring; **c** bract; **d**, **d’** staminate flower; **e**, **e’** stamen; **f**, **f’** carpellate flower; **g**, **g’**, **g”** style and stigma; **h** capsule; **i** cross section of ovary. All from *C.*-*I Peng 23463* (HAST). Line drawing by Che-Wei Lin.

**Figure 5:**
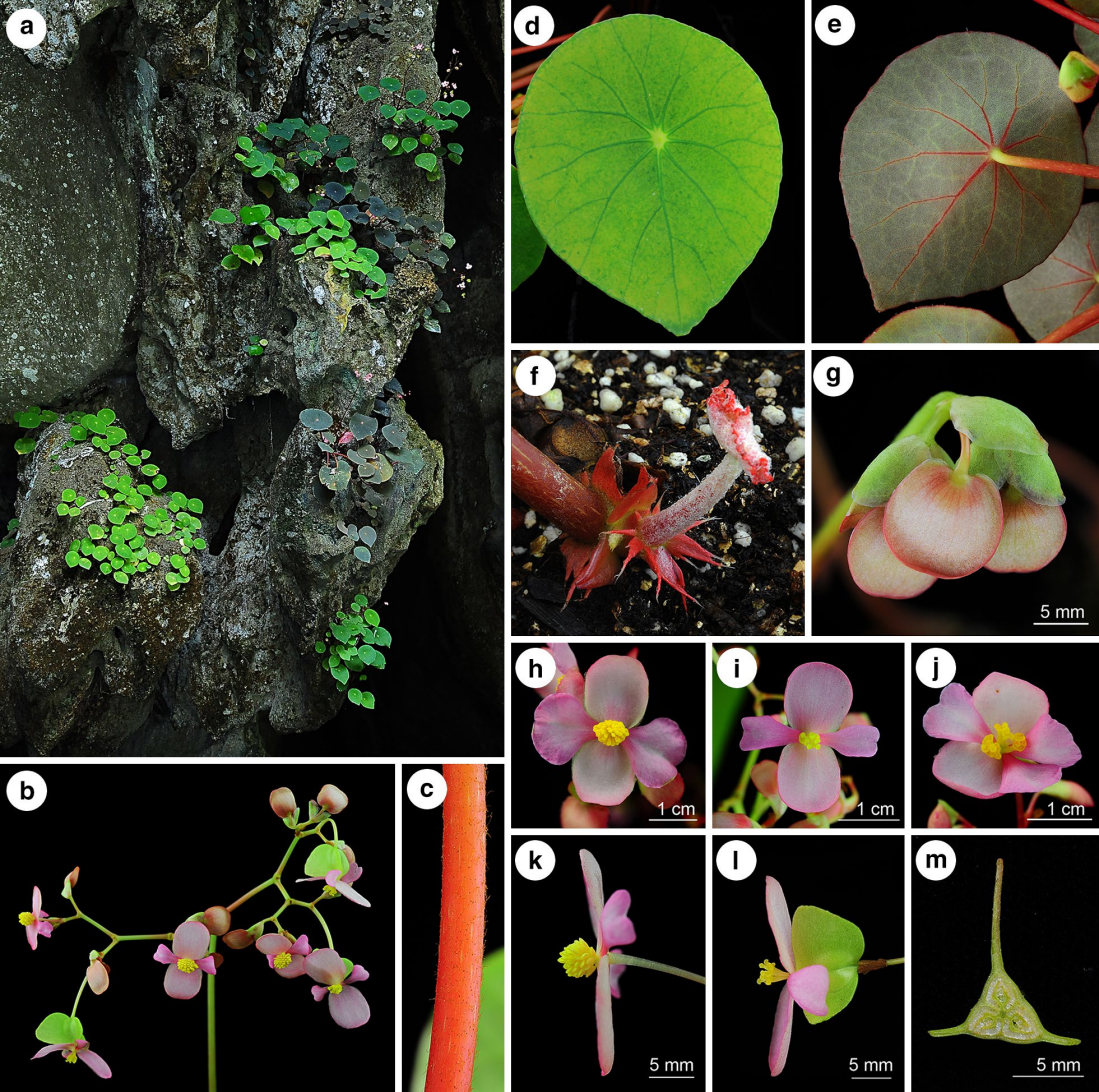
*Begonia taraw* C.-I Peng, R. Rubite & M. Hughes. **a** Habit and habitat; **b** inflorescence; **c** petiole; **d** leaf adaxial surface; **e** leaf abaxial surface, red type; **f** young leaf, stipules and fleshy hairs fused into a ring at the base of the petiole; **g** bracts and young inflorescence; **h** staminate flower, face view; **i** 4-tepaled carpellate flower, face view; **j** 5-tepaled carpellate flower, face view; **k** staminate flower, side view; **l** carpellate flower, side view; **m** cross section of ovary. All from *C.*-*I Peng 23463* (HAST) except E & J from *C.*-*I Peng 23464* (HAST).

1. *Begonia taraw* C.-I Peng, R. Rubite & M. Hughes, sp. nov. § *Baryandra* (Figures 4, 5)

TYPE: PHILIPPINES. Palawan, Puerto Princesa, Puerto Princesa Subterranean River National Park, elev. ca. 5 m, 10°12′1″N, 118°55′32″E, 2 Nov. 2011, *Ching*-*I Peng 23464*, with Kuo-Fang Chung, Chien-I Huang, Rosario Rubite (holotype PNH, isotype E, HAST).

*Begonia taraw* can be differentiated from the only other peltate species in Palawan, *Begonia gutierrezii*, in having longer petioles which are sparsely lanate with appressed hairs (not erect 4 mm long hairs), short internodes on the rhizome (not stoloniferous and elongate), larger leaves, glabrous stipules, and a conspicuous ring of fused fleshy hairs at the base of most petioles.

Lithophytic rhizomatous herb 20–35 cm tall. *Stem* 11–16 mm diameter, glabrous, internodes 5–10 mm long. Stipules broadly triangular, asymmetric, 13–15 × 15–17 mm, keeled, acuminate, entire, glabrous, persistent, maroon becoming dark brown and papery with age. *Leaves* on erect petioles; petioles 13–20 cm long, red at the base, greener apically, terete, 5–7 mm in diameter at the base becoming more slender apically, sparsely lanate when young, the hairs appressed, subglabrous with age, the hairs rubbing off easily, broad-based 10 mm long fleshy red hairs fused into a ring at the base of the petiole; lamina peltate, petiole inserted 3–3.5 cm from midrib, distinctly coriaceous, not variegated, in various colour forms on different plants from pale to very dark matt green above, whitish to pinkish green below, broadly ovate, 6–8 × 9–10 cm, upper surface mostly glabrous, sometimes with a few scattered 2 mm fine long brown hairs between the veins, the pale eye above the petiole attachment with a few hairs; lower surface sparsely lanate on the veins and lamina; venation palmate, main veins 9, pink or green, slightly prominent beneath, flat above; apex obtuse; margin very shallowly sinuate-dentate; edges reddish becoming green when mature; stomata in clusters of 3–7. *Inflorescence* erect, 40–58 cm long, bisexual, male and female flowers open at the same time as the inflorescence matures, cymose, each unit consisting of a central male flower with a lateral female and lateral further unit, branching up to 6 times, with 40–80 flowers in total, not mass-flowering; primary peduncle 30–45 cm long, 5–7 mm diameter at the base becoming more slender apically, base is reddish becoming green at the upper third part, sparsely lanate when young, subglabrous with age, the hairs rubbing off easily, secondary peduncles 1.5–4 cm long, subglabrous; bracts broadly rounded-triangular, conduplicate, ca. 10 × 12 mm, entire, glabrous, deciduous, overlapping, translucent pale green or pinkish green. *Male flowers* pedicel 8–15 mm long, glabrous; tepals 4, uniformly white or pink, entire, glabrous; outer 2 oblong, 13–17 × 10–13 mm; inner 2 obovate, folded and retuse, 11–14 × 8–9 mm; androecium with ca. 50 stamens, sessile; filaments united at the base, unequal, 1.25–2 mm long, longer filaments found in the centre of the androecium; anthers yellow, 1.75 mm long, oblong-oblanceolate, slits lateral tending to unifacial towards the apex, running for 3/4 the length of the anther, connective extended. *Female flowers* pedicel 20–30 mm long, glabrous, with 2 min fleshy 0.5 mm long hair-like bracteoles spaced 1–2 mm below the ovary; ovary 12 × 20 mm including wings, green or pink; capsule ca. 9 × 6 mm, 3-locular, placentae bifid; wings 3, unequal, larger wing ca. 9 × 14 mm, shallowly angular to rounded, flat (not cucullate), smaller 2 wings ca. 11 × 4 mm, truncate; tepals the same as the male flowers; styles 3, bifid, stigmatic surface spirally twisted. *Fruit* drying pale brown, the same size and shape as the ovary. Somatic chromosome number, 2*n* = 28.

*Distribution, habitat and ecology* *Begonia taraw* is currently known only from the type locality and a further field observation from Lions Cave ca. 500 m away. The species grows on vertical limestone cliffs semi-shaded by broadleaf forest at the entrance of the Puerto Princesa underground river. The species was flowering and fruiting when collected in early November. In cultivation in the greenhouse of Academia Sinica in Taipei, Taiwan, it flowered and fruited from July to December.

*Etymology* The species is named after the local word “taraw” which means karst limestone in Tagbanua language.

*Additional specimen examined (paratype)* PHILIPPINES. Palawan, Puerto Princesa Subterranean River National Park, elev. ca. 5 m, 10°12′1″N, 118°55′32″E, 2 Nov. 2011, *Ching*-*I Peng 23463*, with Kuo-Fang Chung, Chien-I Huang, Rosario Rubite (HAST).

*Proposed IUCN red list category* VUD2.The species is a rare, very narrow endemic, and as such has an area of occupancy and extent of occurrence under the threshold for Critically Endangered, (IUCN 2012). However, none of the other criteria, based on number of locations and decline or fluctuation in range or population apply. *B. taraw* is best considered as belonging to the Vulnerable category, under VUD2, a category often applied to naturally rare and narrowly endemic taxa which, although not under immediate threat, have such a narrow range that unpredictable localised habitat degradation puts them at risk of rapidly fulfilling the criteria for Critically Endangered or Extinct.

**Figure 6:**
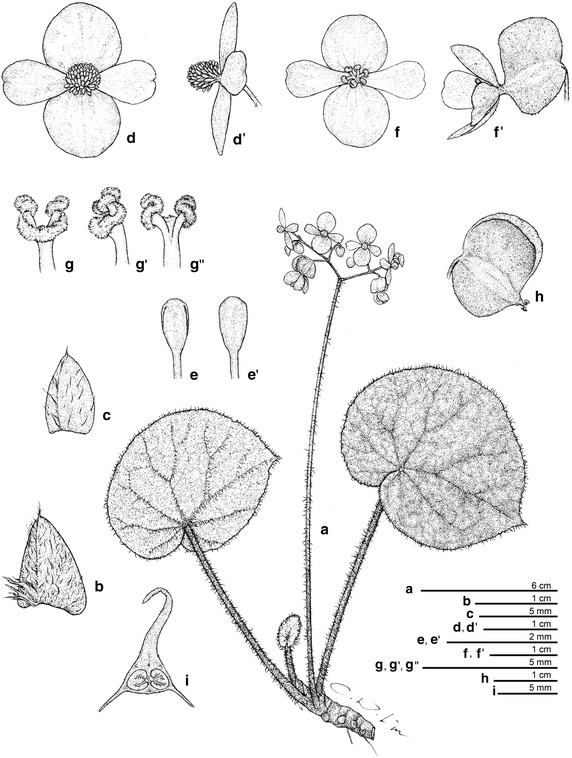
*Begonia hughesii* R. Rubite & C.-I Peng. **a** Habit; **b** stipule; **c** bract; **d**, **d’** staminate flower; **e**, **e’** stamen; **f**, **f’** carpellate flower; **g**, **g’**, **g”** style and stigma;** h** capsule;** i** cross section of ovary. All from *C.*-*I Peng 23466* (HAST). Line drawing by Che-Wei Lin.

**Figure 7:**
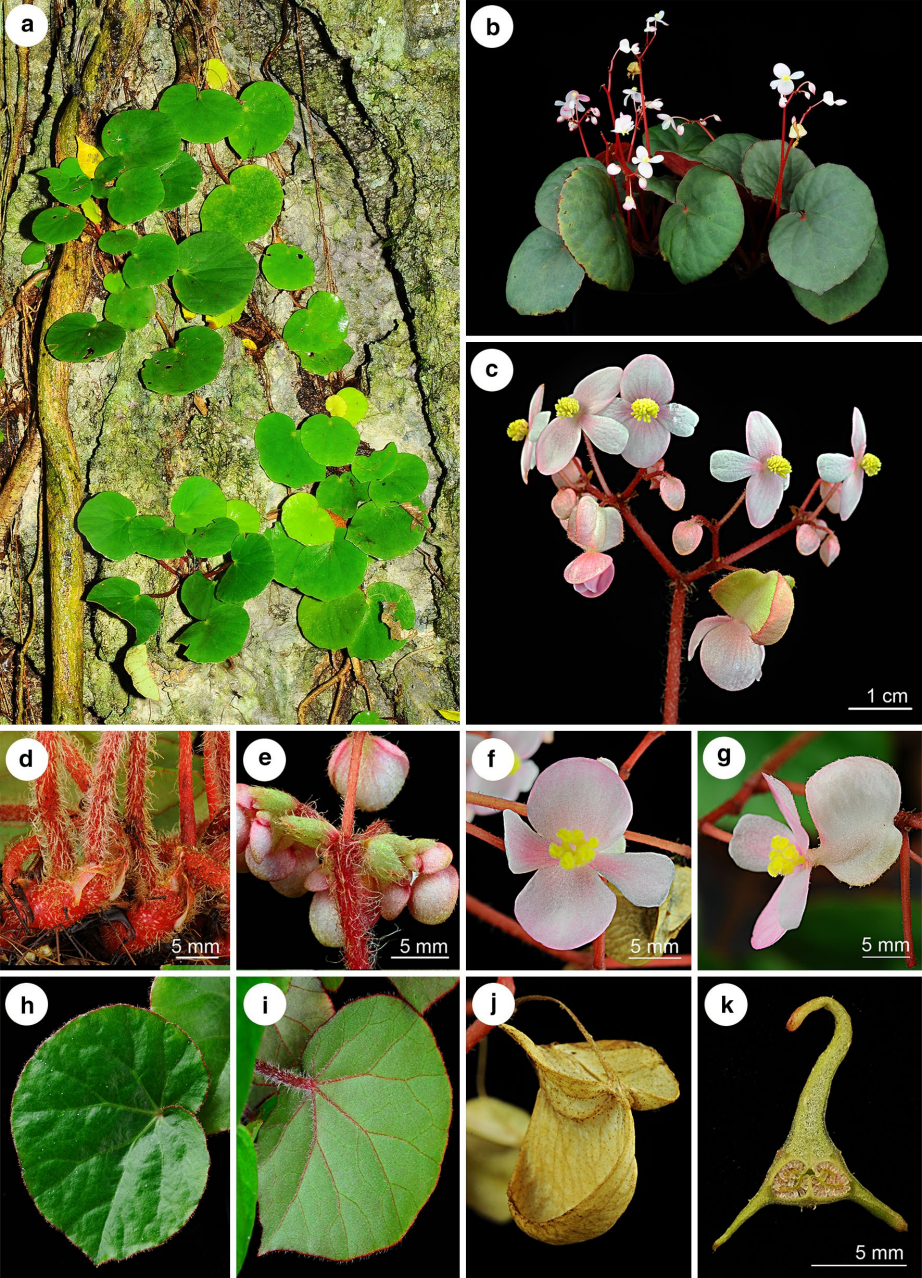
*Begonia hughesii* R. Rubite & C.-I Peng. **a** Habit and habitat; **b** cultivated plant at anthesis; **c** inflorescence; **d** rhizome, stipules and petioles; **e** bracts and young inflorescence; **f** carpellate flower, face view; **g** carpellate flower, side view; **h** leaf adaxial surface; **i** leaf abaxial surface; **j** capsule; **k** cross section of ovary. All from *C.*-*I Peng 23466* (HAST).

2. *Begonia hughesii* R. Rubite & C.-I Peng, sp. nov. § *Baryandra* (Figures 6, 7)

TYPE: PHILIPPINES. Palawan Island, Palawan Province, Puerto Princesa City, Puerto Princesa Subterranean River National Park, elev. ca. 5 m, 10°12′1″N, 118°55′32″E, 2 Nov. 2011, *Ching*-*I Peng 23466*, with Kuo-Fang Chung, Chien-I Huang, Rosario Rubite (holotype PNH, isotype HAST).

*Begonia hughesii* is most similar to *B. acclivis*, but differs in having leaves which are glabrous above (not with scattered 2 mm long hairs) and uniform bright green (not markedly variegated), and dimorphic stipules which are either hairy on the keel only or hairy all over the abaxial surface.

Lithophytic rhizomatous herb ca. 20 cm tall. *Stem* 5–10 mm thick, tomentose (2–3 mm brown hairs) becoming glabrous in parts with age, internodes 3–8 mm long. Stipules triangular, asymmetric, 10–12 × 9–11 mm, heteromorphic, alternately either with brown hairs on the keel only, or tomentose all over abaxially, hairs brown, 2–3 mm long, keeled, keel extending at the tip for ca. 2 mm, ring of hairs at base of petiole absent. *Leaves* on erect petioles; petioles 6–15 cm long, 4–6 mm in diameter, terete, red, tomentose even when mature and with 2 hair types, one linear, the other thicker towards the base; lamina basifixed, thick and fleshy in life, drying opaque and stiff, ovate, 7–10 × 5–7.5 cm, apple-green above, pale green below, glabrous above except for minute pubescence near the petiole attachment, below hairy on the veins only with ca. 2 mm long hairs, venation palmate, main veins 7; margin maroon, broadly and shallowly undulate, dentate-sinuate, ciliate with pink hairs (1–1.5 mm); tip shortly acuminate; stomata in clusters of 2–5. *Inflorescence* erect, ca. 20 cm long, bisexual, male and female flowers open at the same time as the inflorescence matures, cymose, branching 4 times; primary peduncle ca. 15 cm long, with scattered hairs (much less dense than on the petioles), bracts small, ovate, 1.5–2 × 1.5–2 mm, with minute scattered glandular hairs. *Male flowers*: pedicel 7–10 mm long, with sparse minute glandular hairs; tepals 4, uniformly very pale pink or white, entire, with a few minute glandular hairs adaxially; outer 2 sub-orbicular 8–9 × 6–7 mm; inner 2 obovate, slightly retuse, 6 × 3 mm; androecium with ca. 40 stamens, sessile; filaments free, unequal, 1–1.75 mm long, longer filaments found in the centre of the androecium; anthers yellow, 1.25 mm long, oblong-oblanceolate, slits lateral becoming unifacial at the apex; connective extended. *Female flowers*: pedicel 7–10 mm long, with sparse glandular hairs; ovary 10 × 14 mm including wings, pink; capsule 9 × 5 mm, two-locular, placentae bifid; wings 3, unequal, larger wing cucullate, smaller wings rounded; tepals the same as in the male flowers; styles 3, stigmatic surface spirally twisted. *Fruit* pedicel 7–12 mm, drying pale brown, 9–14 × 14–18 mm, recurved with the 2 lateral wings (9–14 × 3–4 mm, round) forming a splash cup, the larger wing markedly cucullate, 9–14 × 7–9 mm, apex truncate. Somatic chromosome number, 2*n* = 30.

*Distribution, habitat and ecology* *Begonia hughesii* herbarium material has been collected from the type locality near the entrance to the Underground River and on Ugong Rock near Sabang and on limestone outcrops at the base of Mt. St. Paul. Further photographic evidence reveal records from the other side of the Babuyan River at PPURNP and within the forest on Cleopatra’s Needle (Jonah Van Beijnen, pers. comm.). The species grows on eroded limestone in semi-shade under broadleaf forest, and was flowering and fruiting when collected in early November. In cultivation in the greenhouse of Academia Sinica in Taipei, Taiwan, it flowered and fruited from July to December.

*Etymology* The species epithet is named after Dr Mark Hughes of the Royal Botanical Garden Edinburgh, who studies extensively Southeast Asian begonias including Philippine *Begonia.*

*Additional specimens examined (paratypes)* PHILIPPINES. Palawan, Sabang, Ugong Rock, 10°5′13″N, 118°51′16″E, elev. ca. 60 m, in limestone outcrops, semishaded, locally abundant, 2 Nov. 2011, *Ching*-*I Peng 23475*, with Kuo-Fang Chung, Chien-I Huang, Rosario Rubite (HAST); Palawan, Mt. St. Paul, elev. 300–600 m, slope forest on karst limestone, 03-05 May 2012, *Hughes & Tandang MH1726* (HAST); Palawan, Puerto Princesa Subterranean National Park, Sabang, jungle trail between CPS and underground river, elev. 50 m, 29 May 2012, M. Hughes, C. Puglisi, D. Tandang & Julius *CP305* (E, PNH).

*Proposed IUCN Red list category* LC. The species has a fairly wide distribution within the PPURNP and surrounding forests on Mt St Paul and Cleopatra’s Needle, and providing these areas remain well managed *B. hughesii* can be considered as Least Concern.

**Figure 8:**
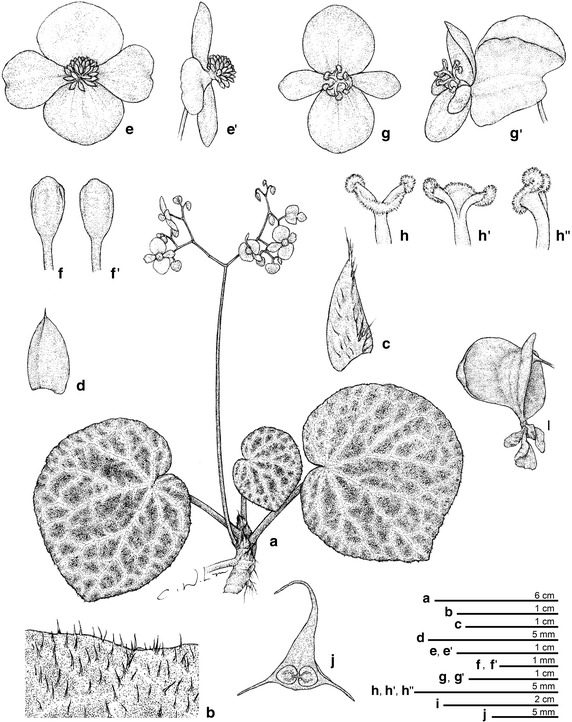
*Begonia tagbanua* M. Hughes, C.-I Peng & R. Rubite. **a** Habit; **b** indumentum on leaf adaxial surface; **c** stipule; **d** Bract; **e**, **e’**, staminate flower; **f**, **f’** stamen; **G**,G’, carpellate flower; **g**, **h**’, **h**”, style and stigma; **i** Capsule; **j** cross section of ovary. All from *C.*-*I Peng 23471* (HAST). Line drawing by Che-Wei Lin.

**Figure 9:**
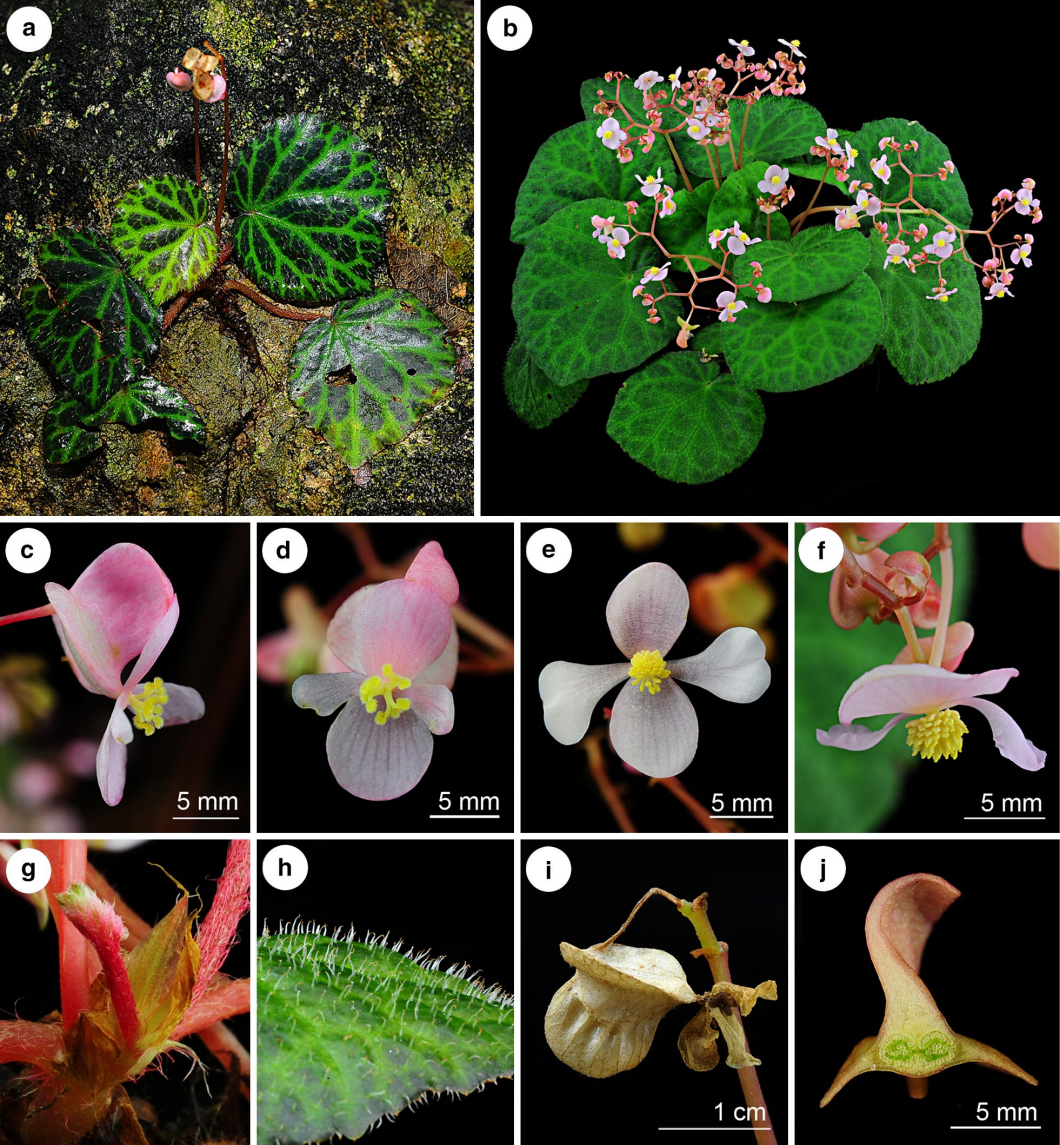
*Begonia tagbanua* M. Hughes, C.-I Peng & R. Rubite. **a** Habit and habitat; **b** cultivated plant at anthesis; **c** carpellate flower, side view; **d** carpellate flower, face view; **e** staminate flower, face view; **f** staminate flower, side view; **g** stipules; **h** leaf adaxial surface, showing indumentum; **i** capsule; **j** cross section of ovary. All from *C.*-*I Peng 23471* (HAST).

3. *Begonia tagbanua* M. Hughes, C.-I Peng & R. Rubite, sp. nov. § *Baryandra* (Figures 8, 9)

TYPE: PHILIPPINES. Palawan, Puerto Princesa, Puerto Princesa Subterranean River National Park, elev. ca. 35 m, 10°12′1″N, 118°55′32″E, 2 Nov. 2011, *Ching*-*I Peng 23471*, with Kuo-Fang Chung, Chien-I Huang, Rosario Rubite (holotype PNH, isotype A, E, HAST, MO).

*Begonia tagbanua* is most similar to *B. suborbiculata*, but differs in having less succulent leaves which are rugolose (not smooth), sparsely puberulous above (not glabrous), and fruits which are 3-winged (not 5-winged).

Terrestrial or lithophytic rhizomatous herb ca. 10 cm tall. *Stem* 6–8 mm in diameter, with sparse 1–2 mm brown hairs becoming glabrous with age, internodes 4–5 mm long. Stipules triangular, 14–16 × 4–5 mm, greenish to pink, with sparse hairs, keel present, apex fimbriate, margin entire. *Leaves* on petioles relaxed against the substrate; petiole 11–13 cm long and 4–6 mm in diameter, terete, succulent, maroon densely covered with brown straight hairs (1–2 mm), simple 2 mm long red hairs in a ring at the base of the petiole; blade ovate to suborbicular; 2 colour forms: one is adaxial surface dark green with brown patches at interveins and abaxial surface maroon, while another is adaxial surface plain apple green and abaxial surface light green; adaxial surface has white straight hairs (2–3 mm) arising from a green or brown dot while abaxial surface has white straight hairs (2–3 mm) at veins and shorter hairs (1–1.5 mm) at interveins, base cordate, lobes rounded, sinus overlap 1 cm, apex obtuse, margin shallowly undulate, denticulate, ciliate with brown straight hairs (1 mm), primary veins 7–8, stomata in clusters of 2–4. *Inflorescence* erect, 22–31 cm long, axillary, arising directly from the rhizome, bisexual, male and female flowers open at the same time as the inflorescence matures, 4 times dichotomously branching; peduncle pink 16–23 cm long, 3–4 mm in diameter, erect, with sparse light brown hairs (1–1.5 mm); bracts rounded-triangular, 4 × 2 mm, glabrous; pedicels pink 1.6–1.8 cm erect to ascending in staminate flowers, 1.5 cm ascending to horizontal in carpellate flowers. Male flowers: pedicel ca. 10 mm, glabrous; tepals 4, pink, glabrous, outer pair orbicular with 10–12 veins; 11–12 × 10–11 mm, inner pair obovate folded and retuse, 8–13 × 7–8 mm; stamens 30–40; filaments 1 mm long, united at the base; anthers yellow, rounded, 0.5 mm long. Female flowers: pedicel ca. 15 mm, ovary 10 × 14 mm including wings, pink; capsule 9 × 4 mm, two-locular, placentae bifid; wings 3, unequal, larger wing cucullate, smaller wings rounded; tepals 4, pink, glabrous, outer pair suborbicular 13–16 × 10–12 mm; inner pair obovate, folded, retuse 11–13 × 4–6 mm; styles 3, stigmatic surface spirally twisted. *Fruit* pedicel 1–1.3 cm; capsule rounded in outline, glabrous, 8–12 × 18–20 mm unequally 3-winged, adaxial wing strongly cucullate shorter than the capsule 7–10 × 8–11 mm; lateral wings 8–12 × 5–6 mm forming a splash cup. Somatic chromosome number, 2*n* = 30.

*Distribution, habitat and ecology* *Begonia tagbanua* is currently known from the type locality in PPSRNP, and from the foothills at the base of Mt. St. Paul at ca. 200 m altitude (M. Hughes field observation). In PPSRNP it grows quite abundantly along trail sides in bare soil, under semi-shaded broadleaf forest. Away from trails it can be found growing on steep banks of bare soil such as can be found at the base of tree roots. The species was flowering and fruiting when collected in early November. In cultivation in the greenhouse of Academia Sinica in Taipei, Taiwan, it flowered and fruited from July to December.

*Etymology* The species is named after the Tagbanuas, one of the indigenous peoples of the type locality.

*Additional specimens examined (paratypes)* PHILIPPINES. Palawan, Puerto Princesa, Puerto Princesa Subterranean River National Park, elev. ca. 35 m, 10°12′1″N, 118°55′32″E, 2 Nov. 2011, *Ching*-*I Peng P23472*, with Kuo-Fang Chung, Chien-I Huang, Rosario Rubite (HAST); Palawan, Puerto Princesa Subterranean National Park, Sabang, jungle trail between CPS and underground river, elev. 50 m, 10°11′53″N, 118°55′5″E, 29 May 2012, M. Hughes, C. Puglisi, D. Tandang & Julius *CP301* (E, PNH).

*Proposed IUCN Red list category* LC. In common with *B. hughesii* and *B. taraw*, *B. tagbanua* is a narrow endemic restricted to the PPSRNP environs. However it is not an obligate lithophyte, and can colonise recently dug clay soil banks by trail sides, and indeed the largest population observed was along the coastal trail toward the subterranean river cave entrance. Hence the appropriate conservation category for the species is Least Concern.

## Discussion

The variety of habitats in terms of forest types and substrates, and in particular the variety of limestone habitats, may play a part in promoting species richness at this relatively low altitude for *Begonia* (Chung et al. 2014). Further exploration towards the karst summit of the nearby Mount St. Paul did not yield any more *Begonia* species, contrary to what we would expect for this predominantly montane genus. The phylogenetic results show that all three species harbour chloroplast polymorphism, which is congruent with *Begonia* species existing as populations with a high degree of genetic isolation (Hughes and Hollingsworth 2008; Twyford et al. 2014). Both *B. tagbanua* and *B. taraw* resolved as monophyletic in the chloroplast phylogeny, whereas *B. hughesii* appears as polyphyletic. Natural hybrids have been reported a number of times in *Begonia* (Peng and Chiang 2000; Peng and Ku 2009; Peng et al. 2010) and it is possible that *B. hughesii* and *B. tagbanua* share a common chloroplast lineage due to a past hybridisation event.

## Conclusions

The study further highlights the importance of the Puerto Princesa Subterranean River National Park as a remarkable World Heritage Site, with its combination of unique geology and endemic flora. The presence of three narrowly endemic and rare species in the most heavily visited part of the reserve means continued care must be taken to ensure trails are well maintained and that they are respected by visitors, and that visitor numbers are managed appropriately. Banks by trail sides can provide an ideal habitat for *Begonia*, particularly if the understory vegetation along the trail is left as intact as possible to provide shade and preserve the micro-climate. Ex-situ conservation collections also have a role to play in ensuring a secure future for the three species described here, giving the potential for re-introduction if a chance event such as drought or typhoon should destroy or drastically reduce the wild populations.

The fact that three new species were found in such close proximity demonstrates how much remains to be discovered in the PPSRNP, and how even small scale forest disturbance could result in extinctions. The study supports the formation of a larger forest reserve encompassing both PPSRNP and the adjacent Cleopatra’s Needle Mountain Range, which also has two recently discovered endemic *Begonia* (Hughes et al. 2010), and doubtless many other endemic plant species awaiting description.

## Additional file

**Additional file 1.** Voucher specimen details and genbank accession numbers for the DNA sequences used in the phylogenetic analysis.
